# Imaging findings of the orbital and intracranial complications of acute bacterial rhinosinusitis

**DOI:** 10.1007/s13244-015-0424-y

**Published:** 2015-08-08

**Authors:** J. W. Dankbaar, A. J. M. van Bemmel, F. A. Pameijer

**Affiliations:** Department of Radiology (HP E01.132), University Medical Center Utrecht, PO Box 85500, 3508 GA Utrecht, The Netherlands; Department of Otorhinolaryngology-Head and Neck Surgery, University Medical Center Utrecht, Utrecht, The Netherlands

**Keywords:** Paranasal sinuses, Acute bacterial rhinosinusitis, Cellulitis, Osteomyelitis, Abscess

## Abstract

**Abstract:**

In patients with acute bacterial rhinosinusitis severe orbital and intracranial complications can occur. This review will illustrate the anatomic relationship between the paranasal sinuses and the orbital and intracranial compartments. Subsequently, the spectrum of orbital and intracranial complications of rhinosinusitis and related imaging findings will be discussed and illustrated by case material from daily practice.

***Teaching Points*:**

*• Acute bacterial rhinosinusitis can cause severe orbital and intracranial complications.*

*• If orbital or intracranial complications are suspected, cross-sectional imaging is mandatory.*

*• Infection can spread from the ethmoid sinus to the orbit through the lamina papyracea.*

*• Frontal sinusitis can spread intracranially through dehiscences or osteomyelitis.*

*• Radiologists must recognize imaging findings of complications of acute bacterial rhinosinusitis.*

## Introduction

Acute bacterial rhinosinusitis frequently evolves from a viral upper respiratory infection (URI). Of all children seeking medical attention for respiratory symptoms, 6 % -7 % have acute bacterial rhinosinusitis [1]. The most common bacterial agents causing this infection are streptococcus pneumoniae, haemophilus influenza and moxarella catarrhalis. Early identification of children with complications of acute bacterial rhinosinusitis is crucial since it can cause life-threatening illness by the spread of infection to the orbits and central nervous system. In clinical practice, orbital complications are encountered most frequently [2]. These typically occur in otherwise healthy young children (age < 5 years) presenting with acute ethmoiditis. If left untreated, orbital complications can result in permanent blindness of the affected side [3]. Clinical symptoms include a swollen eye with or without proptosis or impaired function of the extraocular muscles (i.e. gaze impairment). Intracranial complications are less common but have a higher morbidity and mortality rate [1]. They typically occur in previously healthy adolescent males presenting with rhinosinusitis in combination with severe headache, photophobia, seizures, or other focal neurologic findings [4]. If there is clinical suspicion of orbital or intracranial complications, cross-sectional imaging of the orbit and brain is mandatory [5].

This review will focus on the complications of acute bacterial rhinosinusitis by firstly reviewing the anatomic relationship of the paranasal sinuses to the orbital and intracranial compartments. Secondly, the various complications and their imaging characteristics will be discussed and illustrated using examples from daily practice.

## Anatomic relationship of the paranasal sinuses to the orbital and intracranial compartments [6, 7]

**Fig. 1:**
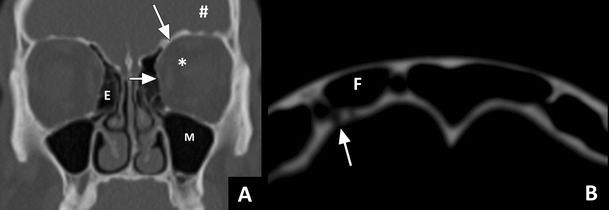
Sinus anatomy: **a**) Coronal CT image in bone window showing the right ethmoid sinus (E); the left maxillary sinus (M); the lamina papyracea (short white arrow); the left orbit (*); the orbital plate (long white arrow); the anterior cranial fossa (#). **b**) Axial image (WL 1500, WW 2600) showing very small congenital dehiscences (arrow) between the frontal sinus (F) and the anterior cranial fossa

**Fig. 2:**
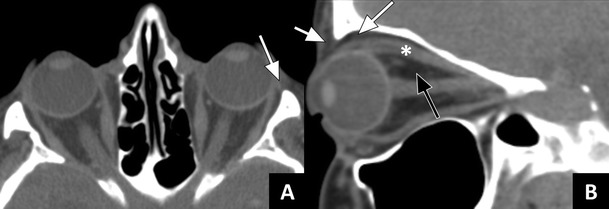
Orbital anatomy: **a**) Axial CT images in soft tissue window showing the orbital septum (arrow). **b**) Sagittal image showing the preseptal fat (short arrow), and the postseptal (extraconal) fat (long arrow); the intraconal fat (black arrow) is separated from the postseptal/orbital fat by the rectus muscles (* superior rectus muscle together with levator palpebrae superioris muscle)

The three most vulnerable anatomic compartments that lie adjacent to the paranasal sinuses are the two orbital and the intracranial compartments (Fig. 1). The ethmoid sinuses are situated between the nasal cavity and the orbit. Cranially, the ethmoid sinuses border the anterior cranial fossa, separated by the skull base, which is a relatively thick barrier. The medial orbital wall is a very thin bony separation between the orbit and the ethmoid sinuses, termed the lamina papyracea. The lamina is not only very thin but also has numerous natural dehiscences and perforating vessels and nerves [8]. Therefore, infection can easily spread from the ethmoid sinus to the orbit. On the orbital side of the lamina there is a periosteal layer termed the periorbita, which serves as barrier against the (early) spread of disease. Infectious spread from the ethmoid sinus to the orbit therefore initially results in subperiosteal abscess formation before spreading to the orbit itself [9]. In the orbit, intraorbital fat lies directly adjacent to the periorbita. Anteriorly, the intraorbital fat and the other structures within the orbit are separated from the extraorbital structures by the orbital septum (Fig. 2). This septum, also termed the palpebral ligament, arises from the periosteum of the orbital rim, and inserts into the superior levator palpabrae, and the lower edge of the tarsal plate. It forms a barrier that prevents spread of infection into the orbit [10]. For this reason, infectious processes around the eye are often classified in relation to the orbital septum as either being preseptal, only involving the eyelid, or postseptal, involving the structures of the orbit. Within the orbit lies the orbital cone, which contains the eyeball and the rectus muscles surrounded by a fascia (Fig. 2). Intraorbital pathology can, therefore, also be divided in intraconal (within the cone), and extraconal pathology.

The roof of the orbit is formed by the frontal bone (i.e. pars orbitalis or orbital plate). Within this bone, the frontal sinus develops. Dorsally, the frontal sinus is directly adjacent to the anterior cranial fossa. Like the lamina papyracea, the frontal sinus may have bony dehiscences that can form a direct route for the spread of infection. Three common sites of congenital dehiscence are: 1) behind the trochlear fossa, 2) behind the supraorbital notch, and 3) at the junction of the middle and outer thirds of the sinus floor (Fig. 2). In addition, acquired dehiscences can exist, for example due to trauma [11].

Besides the spread of infection through the described dehiscences and canals there are two other mechanisms of spread that play an important role in the complications of bacterial rhinosinusitis. Firstly, there can be a bacterial thrombophlebitis through valveless veins causing spread of infection to the cavernous sinus [8, 12]. Secondly, there can be direct extension of infection through osteomyelitis [12].

## Complications of acute bacterial rhinosinusitis and their imaging characteristics

### Imaging considerations

The transition from uncomplicated URI to acute bacterial rhinosinusitis is not straightforward. There is considerable overlap between the symptoms and clinical findings of both entities. Imaging has not been shown to be helpful in distinguishing acute bacterial rhinosinusitis from viral URI. In children younger than 3 years with untreated, uncomplicated, rhinosinusitis or in patients of any age who have an uncomplicated cold for less than 10 days, imaging is not indicated [1, 5]. Especially in the paediatric population soft tissue swelling of the paranasal sinuses on CT and MRI is very common. Extensive mucosal thickening is present in up to 68 % of cases [13, 14]. Generally, rhinosinusitis is a diagnosis based on clinical symptoms. However, distinguishing self-limiting infection from rhinosinusitis with orbital or intracranial complications can be challenging. If complications are clinically suspected contrast-enhanced CT (ceCT) and/or MRI (ceMRI) of the paranasal sinuses should be obtained [1]. To date, there are no head-to-head comparisons of the diagnostic accuracy of ceCT to ceMRI in evaluation of complicated rhinosinusitis in children. Since bony sinus anatomy can be best depicted by CT, CT is often the imaging modality of first choice [15]. In addition CT is widely available, fast, and provides high spatial resolution images. Because of the speed of the acquisition, sedation is usually not required. An important disadvantage of ceCT is the use of ionizing radiation. Especially in children, exposure to radiation should be kept at a minimum. An important disadvantage of ceMRI is that it requires patient sedation in young children. In addition, MRI may not be available around the clock in all hospitals. However, some reports have illustrated that abnormalities responsible for the clinical symptoms are better seen on ceMRI. This seems to be especially true for intracranial complications [16, 17]. In patients that do not require sedation, ceMRI may be preferred over ceCT. If ceCT shows no abnormalities, but the patient has persisting symptoms suspicious for orbital or intracranial complications ceMRI should be obtained [1].

The acquisition of an orbital ceCT at our institution is done with 0.9 mm slice thickness, 0.45 mm increment, 120 kV, 55 mAs, 140 FOV, and a 512 × 512 matrix. The CT is acquired 2 minutes after manual injection of 100 ml of iodinated contrast material. Two mm thick axial and coronal slices are reconstructed using filtered backprojection with a soft tissue kernel. In addition, multiplanar reconstructions can be made in any plane from the thin slice source data.

An MRI protocol of the orbit should comprise at least coronal T2 STIR, axial T1, and axial and coronal T1 SPIR after gadolinium with a slice thickness of 3 mm or less and a pixel size of 1 mm or less [18]. In addition diffusion-weighted imaging (DWI) has shown to be helpful in the evaluation of orbital infection [19].

The MRI protocol for intracranial complications should in addition to contrast enhanced T1 weighted (T1w) images comprise DWI, and fluid attenuated inversion recovery (FLAIR) images. DWI is known to be highly sensitive to pus formation and FLAIR can be helpful in detecting oedema and leptomeningitis [20–22].

### Orbital complications

The orbital complications of rhinosinusitis have been classified by Chandler et al. into five categories: 1) preseptal cellulitis; 2) orbital cellulitis; 3) subperiosteal abscess; 4) orbital abscess; and 5) cavernous sinus thrombosis [23]. Chandler 1 to 3 is primarily treated with intravenous antibiotics. However, if the patient develops loss of vision or shows progressive signs of systemic disease within 24-48 hours, additional functional endoscopic sinus surgery will be performed [24]. Chandler 4 and 5 are directly treated with functional endoscopic sinus surgery and orbital drainage in addition to intravenous antibiotics. In case of cavernous sinus thrombosis anticoagulation therapy can be applied. This is however controversial since intracranial haemorrhage may occur [25].

**Fig. 3:**
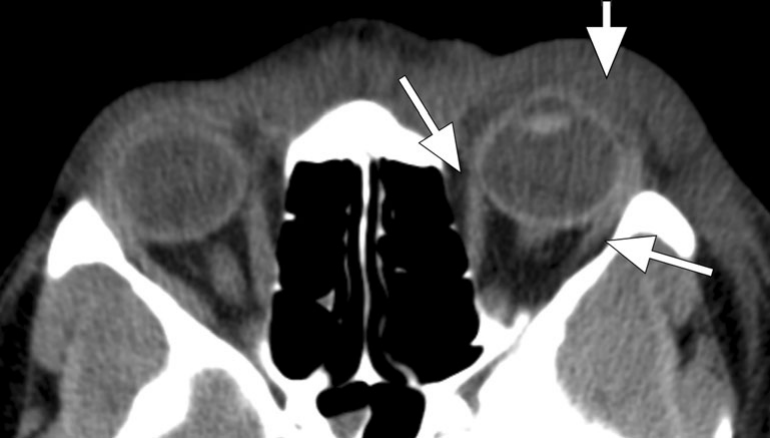
Isolated preseptal cellulitis: Axial ceCT image in soft tissue window showing a thickened and infiltrated eyelid (short arrow); the postseptal fat (long arrows) looks impeccable. Note the absence of signs of infection in the ethmoid sinus

**Fig. 4:**
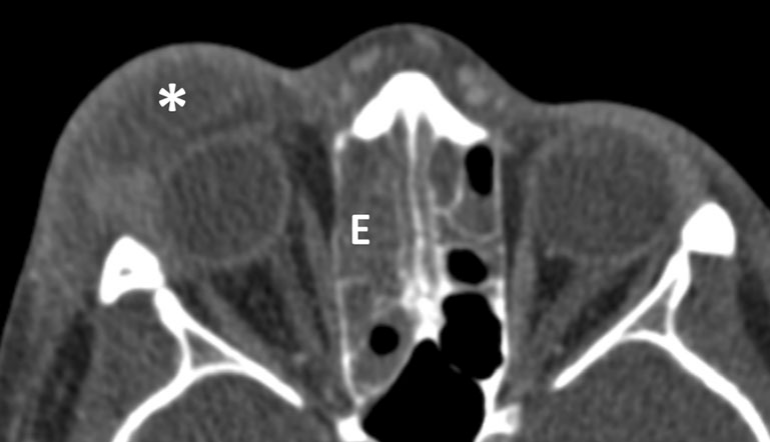
Preseptal abscess: Axial ceCT image in soft tissue window showing a heterogeneous collection in the right eyelid with rim-enhancement (*) indicative of preseptal abscess; note the adjacent opacification of the ethmoid sinus (E) indicative of ethmoiditis

Preseptal cellulitis refers to swelling of the eyelid due to inflammatory oedema anterior to the orbital septum. The eyelid is usually not painful, there is no chemosis, and extraocular muscle movement and vision are not impaired. On CT, preseptal cellulitis is seen as thickening and infiltration of the eyelid (Fig. 3). Formation of a preseptal cellulitis in preseptal abscess is rare [10]. In these cases CT images will show a preseptal fluid collection with a thick enhancing rim (Fig. 4).

**Fig. 5:**
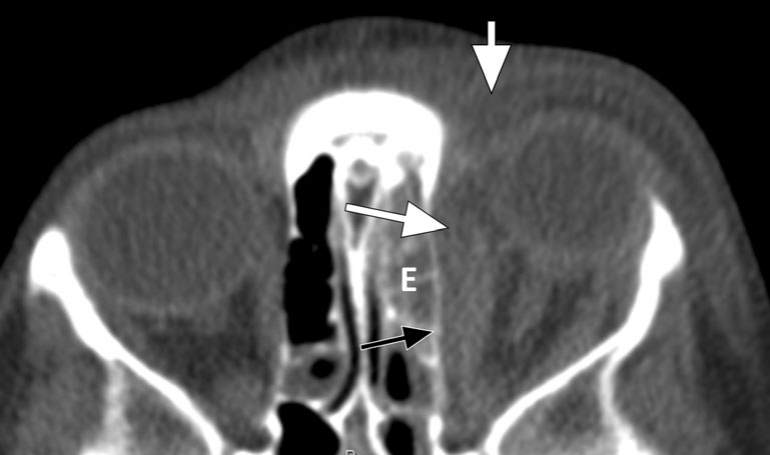
Orbital cellulitis: Axial ceCT image in a soft tissue window showing opacification of the left ethmoid sinuses (E), infiltration and thickening of the eyelid (short white arrow) indicative of preseptal cellulitis, and infiltration of the postseptal/orbital fat (long white arrow) adjacent to the lamina papyracea (black arrow) indicative of orbital cellulitis and possibly early subperiosteal abscess

Orbital cellulitis refers to oedema and inflammation of the orbital contents without abscess formation. Proptosis and impaired eye movement are reliable signs of orbital cellulitis. Also chemosis is usually present. At this stage loss of vision is still rare [8, 10]. The vision should, however, be monitored. On CT, orbital cellulitis results in smudging of the intraorbital fat usually in combination with preseptal cellulitis (Fig. 5). If the CT findings are unclear and orbital cellulitis is still suspected, MRI may be of help. Especially fat saturated T2 weighted MR sequences (like STIR) are highly sensitive to inflammatory changes in the orbit and should be included in every MRI protocol for orbital imaging. In addition, DWI can aid in the differentiation between orbital cellulitis and entities with overlapping imaging characteristics like orbital inflammatory syndrome and lymphoid lesions [26].

**Fig. 6:**
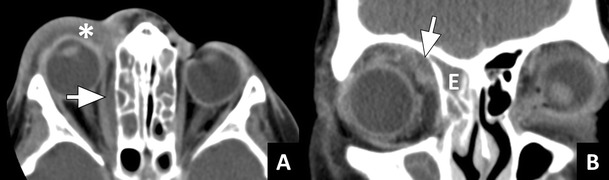
Subperiosteal abscess: **a**) Axial ceCT image in a soft tissue window showing opacified ethmoid sinuses; preseptal cellulitis (*); and a small crescent shaped soft tissue mass (arrow) adjacent to the lamina papyracea, highly suspicious for subperiosteal abscess. **b**) Coronal ceCT image in another patient with opacification of the right ethmoid sinus (E) and a crescent shaped fluid collection adjacent to the medio-superior orbital wall, suspicious for subperiosteal abscess

Subperiosteal abscess refers to abscess formation between the bone, usually the medial or superior orbital wall, and the periorbita. The abscess collection can displace the orbital content, thereby causing proptosis and impaired extraocular muscle movement. Again, chemosis is usually present. Vision is usually not affected [8]. On CT, subperiosteal abscess can be hard to detect especially at an early stage. The orbital fat adjacent to the medial orbital wall should be impeccable. Any soft tissue density at this site in the presence of a fluid-filled paranasal sinus is suspicious for subperiosteal abscess formation [27, 28]. Once the abscess becomes larger it will appear as a fluid-filled crescent shaped mass with rim enhancement. Since the abscess is positioned below the periosteum, the angles between the mass and the orbital wall will typically be obtuse (Fig. 6).

**Fig. 7:**
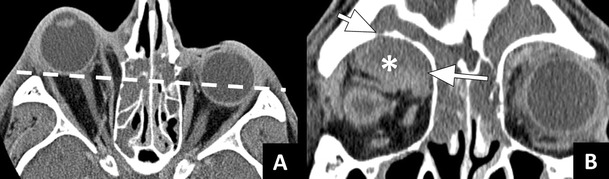
Orbital abscess: **a**) Axial ceCT image in a soft tissue window showing severe right sided proptosis with the entire right eyeball lying anterior to the interzygomatic line (dotted line). **b**) Coronal image in the same patient showing a large intraorbital fluid collection with a thick enhancing rim (*) and sharp angles in relation to the orbital wall (long arrow). These imaging findings are indicative of an orbital abscess. The floor of the frontal sinus shows a bony dehiscence (short arrow)

Orbital abscess refers to pus within the orbital content, resulting from progression of orbital cellulitis or rupture of a subperiosteal abscess. Orbital abscess results in severe proptosis, ophthalmoplegia, and often loss of vision [3, 8]. In patients with bacterial rhinosinusitis the abscess will usually be extraconal. On CT, orbital abscess can be seen as a rim-enhancing fluid collection that has sharp angles with the orbital wall (Fig. 7) [28].

On ceMRI, both subperiosteal and orbital abscesses are seen as T1 isointense, T2 hyperintense fluid collections with rim enhancement. In addition, the presence of pus will result in restricted diffusion on DWI [19].

**Fig. 8:**
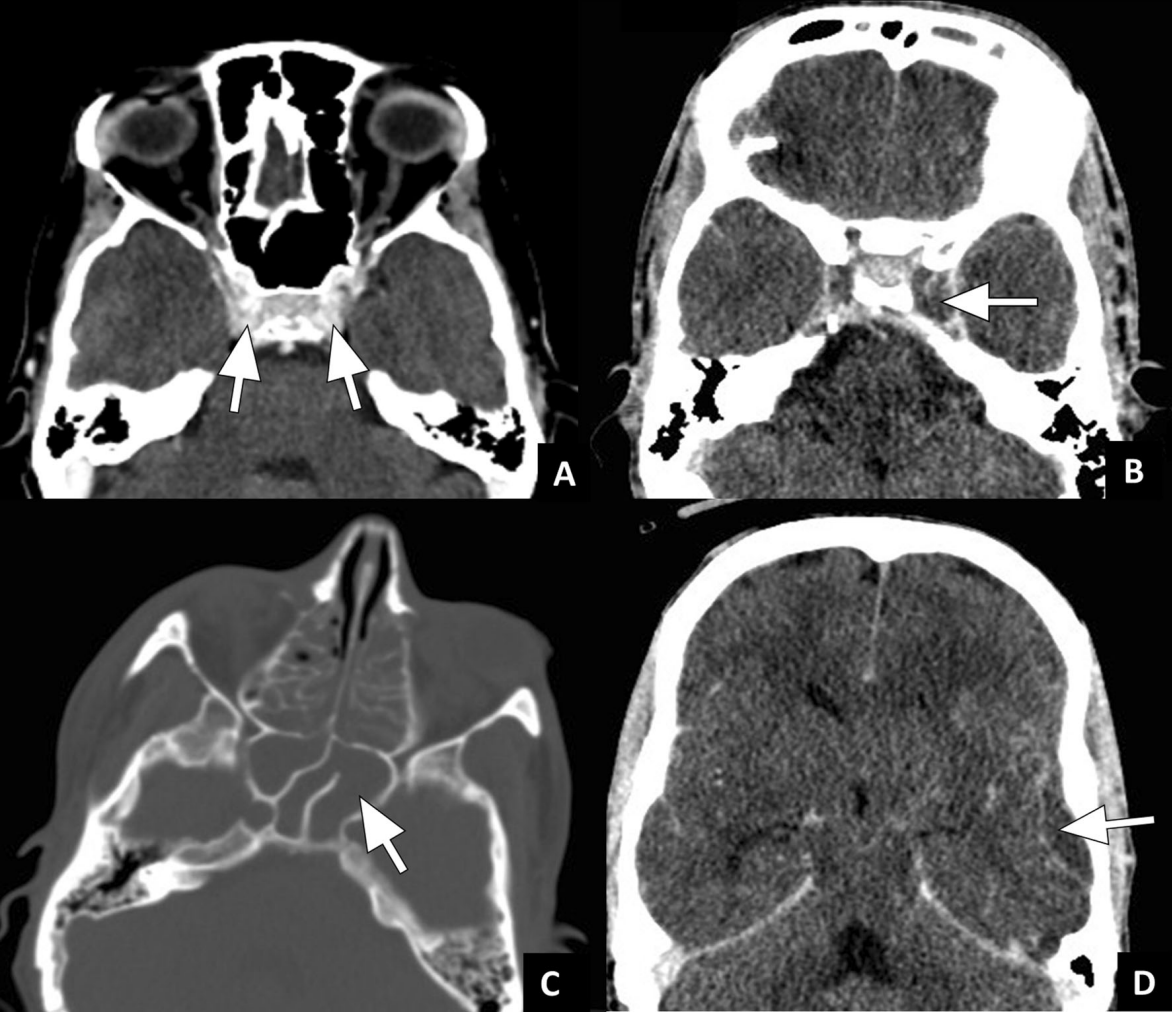
Cavernous sinus thrombosis case 1: **a**) Axial ceCT image in a soft tissue window at the level of the skull base showing normal enhancement of the cavernous sinus (arrows). **b**) ceCT in a different patient showing filling defects in the cavernous sinus with slight enlargement on the left side (arrow), indicative of thrombosis. Axial images at different levels in the same patient showing **c**) opacification of the sphenoid (arrow) and ethmoid sinuses in bone window and **d**) an extradural fluid collection with meningeal enhancement suspicious for empyema in soft tissue window

**Fig. 9:**
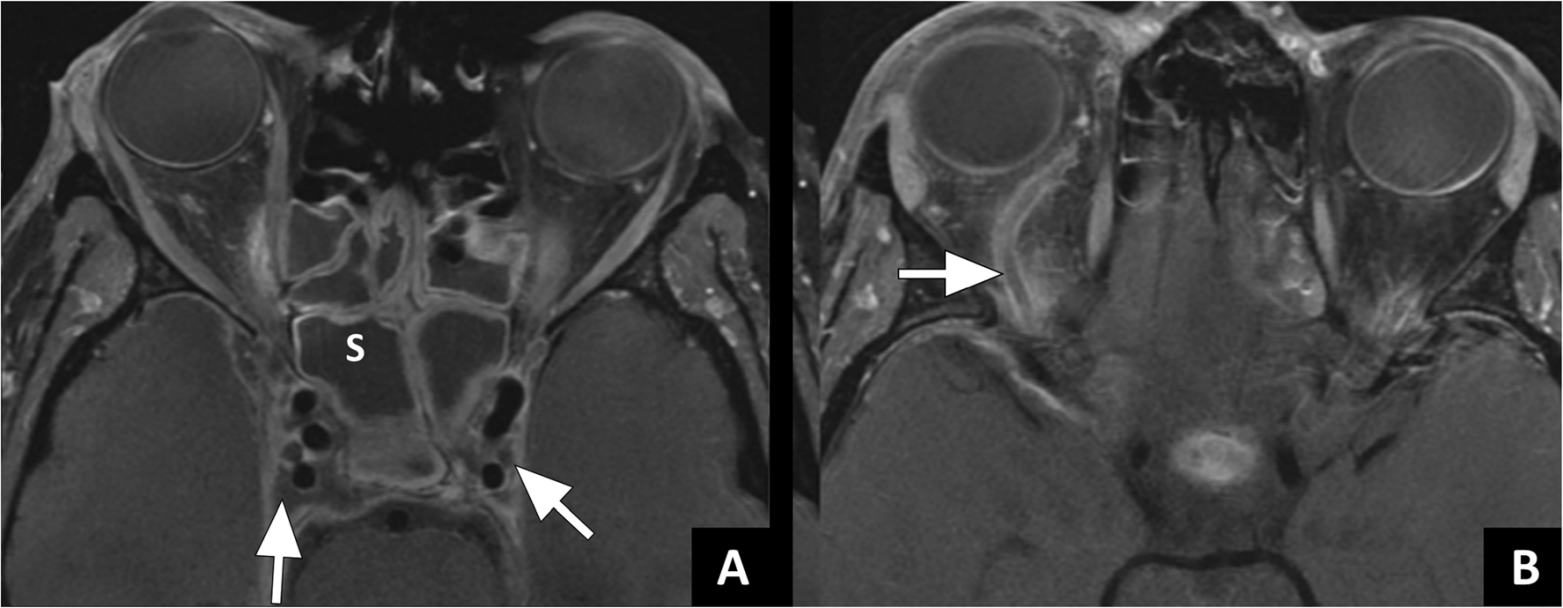
Cavernous sinus thrombosis case 2 (Courtesy P. de Graaf, MD PhD, VU University Medical Center, the Netherlands): **a**) Axial T1w ceMRI showing filling defects bilaterally in the cavernous sinus (arrows) and fluid in the sphenoid sinus. **b**) Axial T1w ceMRI in the same patient showing a filling defect in the right ophthalmic vein (arrow). The findings are indicative of cavernous sinus thrombosis and thrombophlebitis in the ophthalmic vein caused by sphenoiditis

Cavernous sinus thrombosis is both classified as an orbital and intracranial complication [1]. It is a result of infectious spread from the orbit through valveless veins [8, 12]. In patients with cavernous sinus thrombosis the classical symptoms are ptosis, proptosis, chemosis, and cranial nerve palsies [29]. The diagnosis can be made both with ceCT and ceMRI (Figs. 8 and 9). If cavernous sinus thrombosis is bilateral, it can be easily missed. The best clue to the presence of a cavernous sinus thrombosis is a filling defect in the sinus that does not represent fat. Fatty deposition in the cavernous sinus is a normal finding that may be more prominent in obese patients or patients with hypercortisolism [30]. In addition to filling defects, a convex margin of the sinus can be seen as a result of mass effect. Since thrombus can have varying signal intensity on MRI, the diagnosis can be challenging on MRI [31]. Subacute thrombus often has high signal intensity on both T1w as well as T2w images [32]. Susceptibility weighted images are particularly useful in visualizing the paramagnetic blood breakdown products of venous thrombi with very high sensitivity [31]. Some thrombi show diffusion restriction on DWI, this finding is however not very specific [31, 33]. Additional helpful findings on MRI are increased dural enhancement along the lateral borders of the cavernous sinus and ipsilateral tentorium [30]. On both ceCT and ceMRI, dilatation or a filling defect of the superior ophthalmic vein can be another important clue to the diagnosis (Fig. 9).

### Intracranial complications

Besides cavernous sinus thrombosis, intracranial complications that can occur in patients with acute bacterial rhinosinusitis are: subdural empyema, epidural empyema, cerebritis, brain abscess, and meningitis [2]. Patients usually present with severe headache in combination with photophobia, seizures, or other focal neurologic findings [4]. If intracranial complications occur, functional endoscopic sinus surgery is mandatory. In the presence of empyema and brain abscess, neurosurgical intervention is required.

**Fig. 10:**
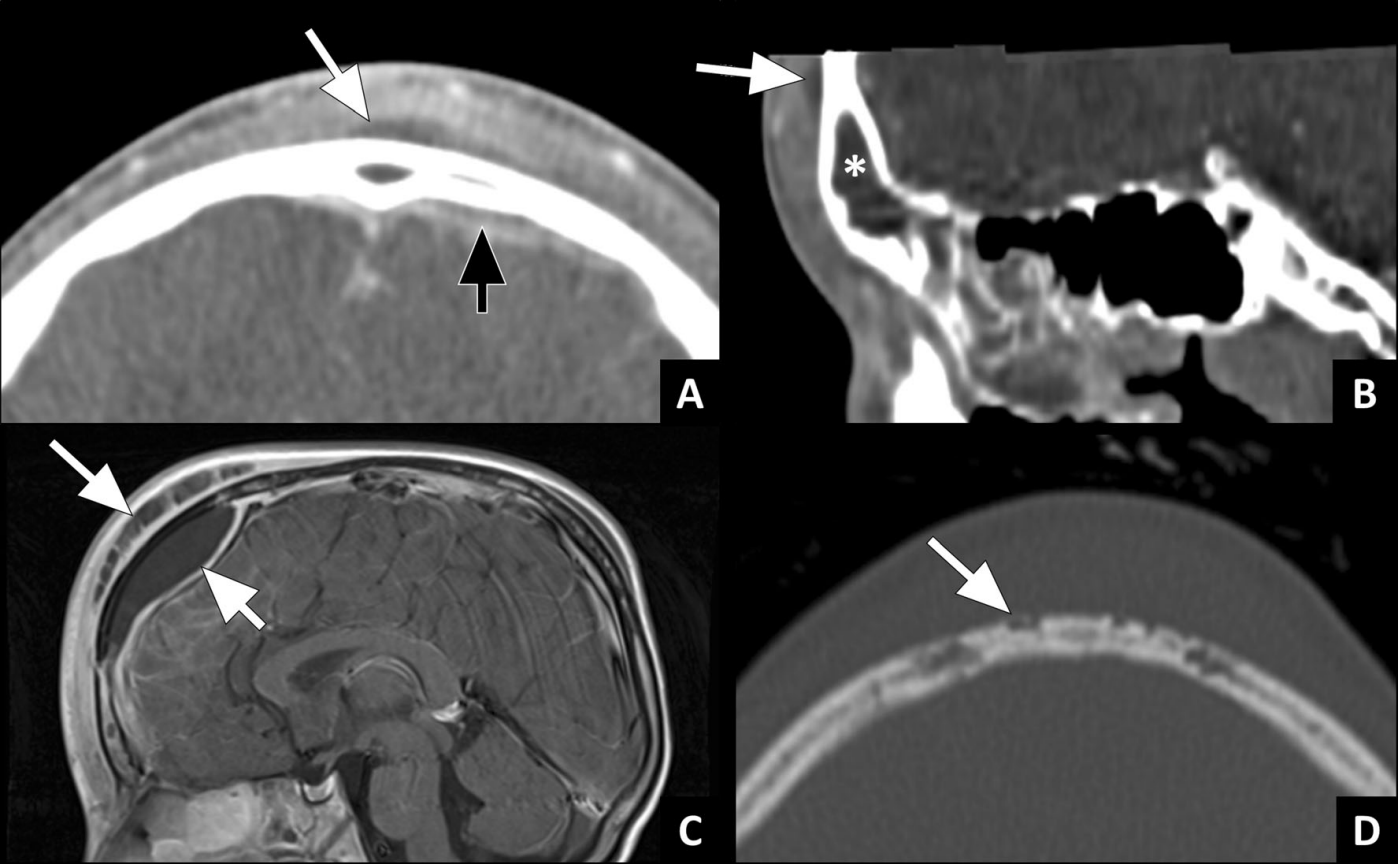
Pott’s puffy tumour: **a**) Axial and **b**) sagittal ceCT image in a soft tissue window showing opacification of the frontal sinus with heterogeneous material (*) and a subperiosteal abscess (white arrow) due to frontal sinusitis. Note the small subdural fluid collection (black arrow) indicating subdural empyema. **c**) Sagittal T1w ceMRI in another patient showing a large subperiosteal abscess (long arrow), and a large extra-axial empyema (short arrow). **d**) Axial CT image in a bone window in the same patient showing erosion of the tabula externa (arrow) adjacent to the soft tissue mass, indicative of osteomyelitis of the frontal bone

Intracranial complications, other than cavernous sinus thrombosis, are often secondary to frontal sinusitis. As mentioned earlier, frontal sinusitis can spread to the anterior cranial fossa directly through bony dehiscences or through osteomyelitis of the frontal bone. In rare cases, osteomyelitis of the frontal bone spreads outward and results in subperiosteal abscess (Pott’s puffy tumour) [34]. The subperiosteal abscess can present with a tender swelling on the forehead in a patient with frontal sinusitis. CT and MRI will show a subperiosteal fluid collection overlying the frontal bone (Fig. 10). Communication between the fluid collection and frontal sinus can usually be seen.

**Fig. 11:**
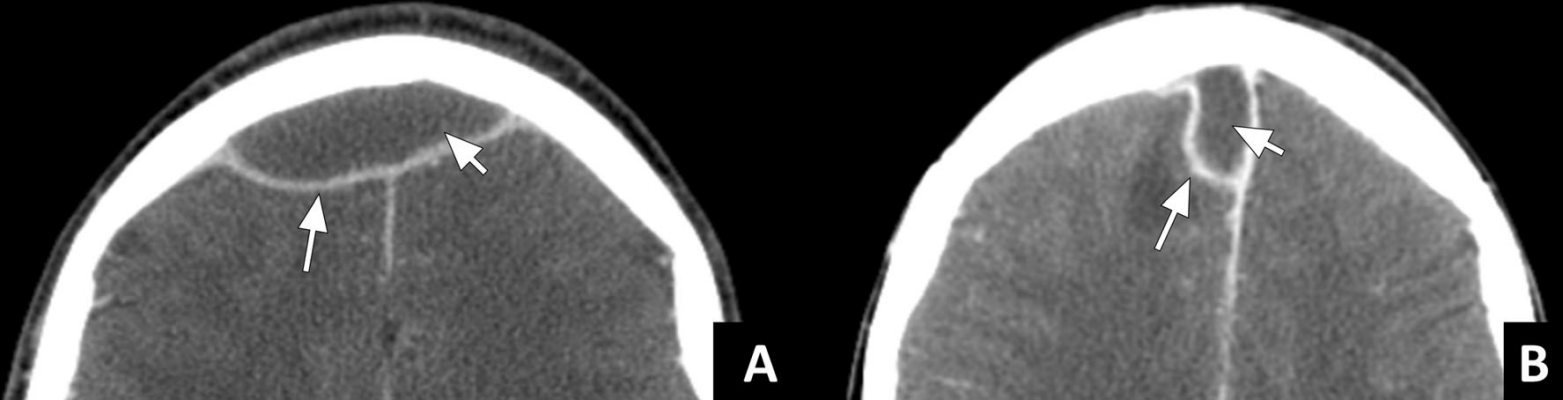
Epidural and subdural empyema: **a**) ceCT in soft tissue window in a patient with frontal sinusitis (not shown) showing an extra-axial fluid collection passing the midline (short arrow) anterior to the falx cerebri. The adjacent meninges are thickened and show strong enhancement (long arrow). These findings are indicative of an epidural empyema. **b**) An extra-axial fluid collection in another patient with frontal sinusitis (not shown) running along the falx cerebri instead of passing it anteriorly (short arrow). Again, the meninges are thickened and show increased enhancement (long arrow). These findings indicate subdural empyema

Infectious spread from the frontal sinus to the anterior cranial fossa primarily results in subdural and epidural empyema. These two entities present as extra-axial fluid collections between the skull and the brain parenchyma. The fluid collections will typically show an enhancing rim. In addition, there will be mass effect on the adjacent brain and reactive oedema [21]. Differentiation between a subdural and an epidural empyema can be difficult. In patients with rhinosinusitis these are often located adjacent to the falx cerebri. If the empyema crosses the falx anteriorly, this is indicative of an epidural location (Fig. 11a). If it stays on one side of the falx and even runs posteriorly along the falx, it lies subdurally (Fig. 11b). On MRI, pus containing fluid collections will show diffusion restriction on DWI allowing differentiation from hygroma [20].

**Fig. 12:**
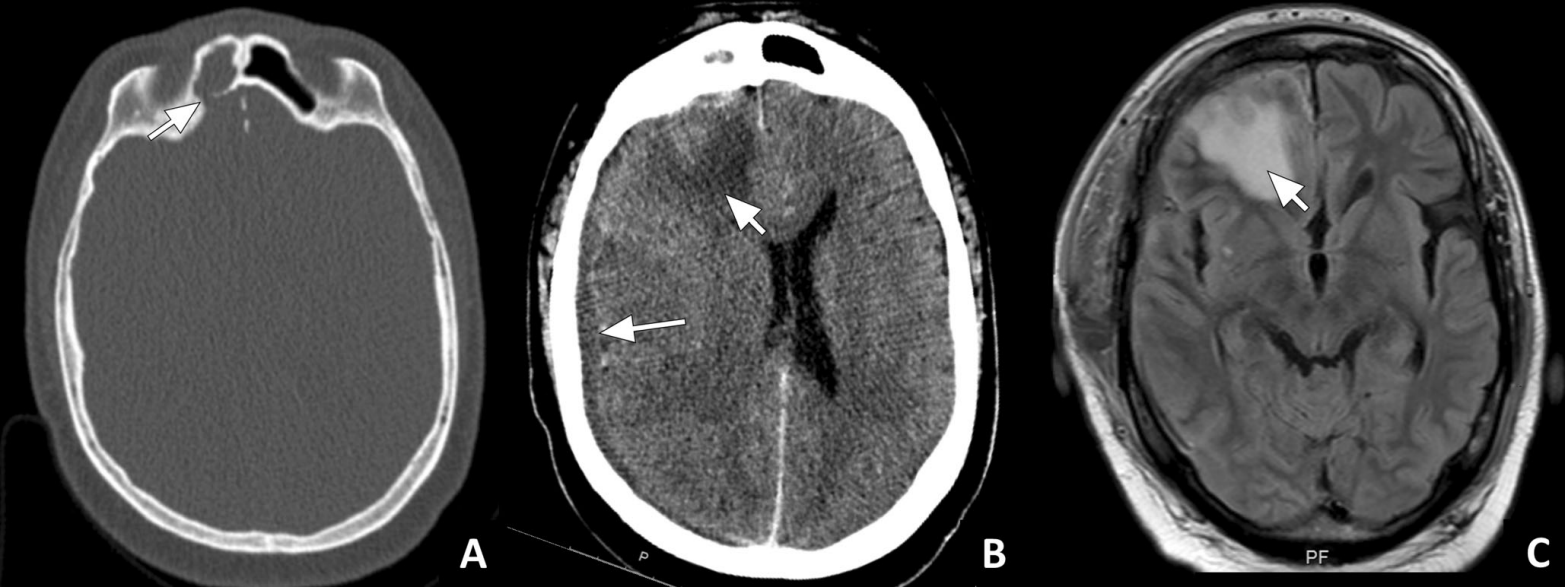
Focal cerebritis: **a**) Axial CT in bone window showing an opacified right frontal sinus with a bony defect in the posterior sinus wall (arrow). **b**) ceCT image in a soft tissue window in the same patient showing a focal area of hypodensity in the right frontal lobe (short arrow) with faint enhancement in the centre. In addition, a subdural fluid collection can be seen (long arrow). **c**) Axial MR FLAIR image of the same patient showing an area of hyperintensity in the left frontal lobe (short arrow). These findings indicate frontal sinusitis with bony erosion causing subdural empyema and focal cerebritis

**Fig. 13:**
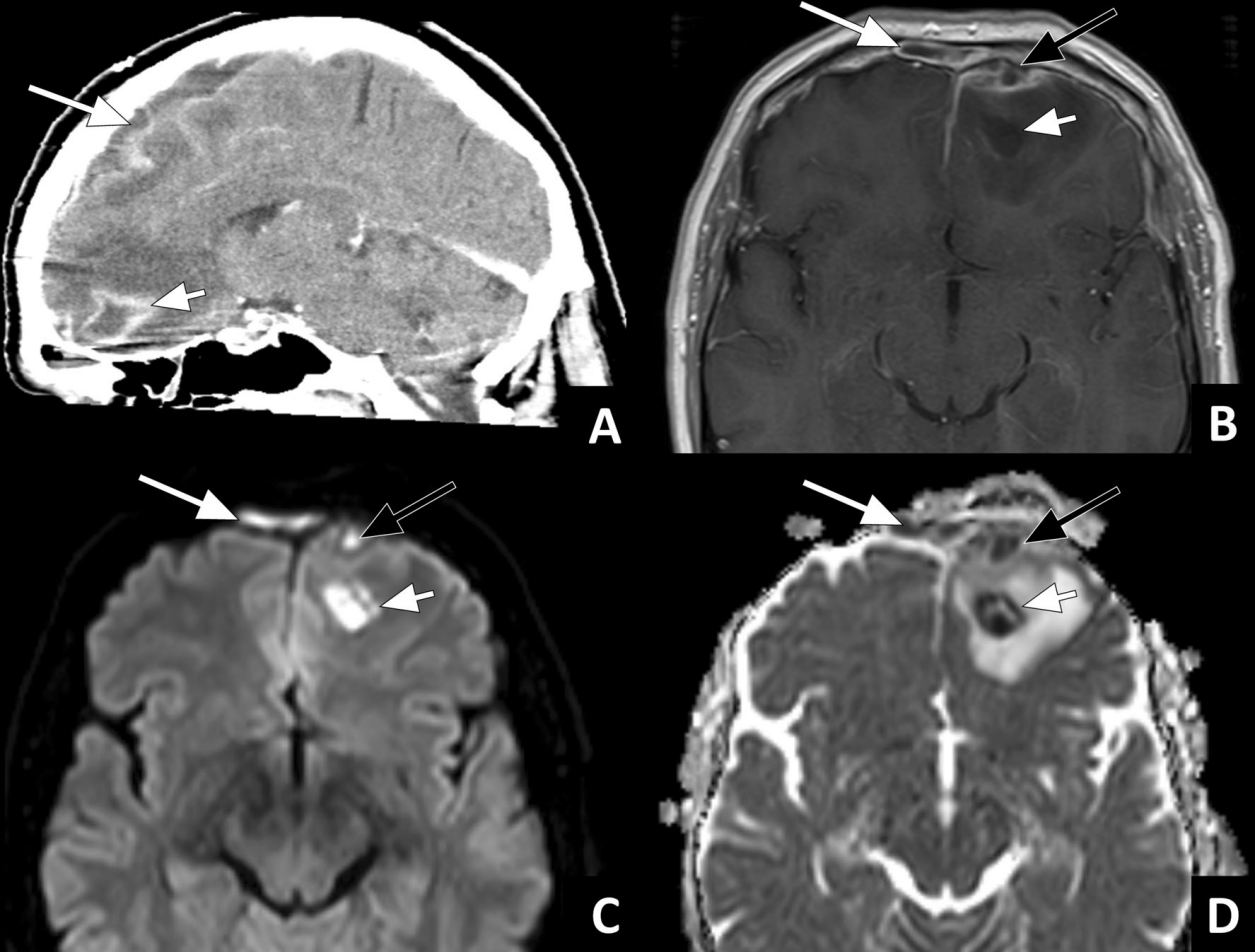
Brain abscess: **a**) Sagittal ceCT image showing an intra-axial fluid collection in the frontal lobe with rim enhancement (short arrow) and surrounding oedema. In addition, a subdural collection with thickening and enhancement of the meninges is present (long arrow). **b**) Axial T1w ceMRI, **c**) DWI b1000, and **d**) ADC map in the same patient showing a fluid collection in the frontal sinus with mucosal enhancement and restricted diffusion (long white arrow), a subdural fluid collection with thickened, enhancing meninges, and restricted diffusion (black arrow), and a fluid collection with rim enhancement in the left frontal lobe with restricted diffusion (short white arrow) and surrounding oedema. The findings are indicative of frontal sinusitis with pus formation, subdural empyema and brain abscess

If the infection spreads from the extra-axial space to the brain parenchyma, focal cerebritis and later brain abscess can develop. At an early stage, this can be hard to detect with CT. The ceCT may only show focal hypodensity with mass effect (Fig. 12a) and sometimes patchy enhancement. T2w sequences and FLAIR are more sensitive in detecting focal oedema (Fig. 12b) and early abscess formation, which is seen as increased signal intensity [21]. If a full blown abscess has formed, a fluid collection with a well-defined enhancing rim with mass effect and pronounced swelling of the surrounding brain parenchyma can be seen both on CT and MRI (Fig. 13). Similar to extra-axial pus collections, pus within a brain abscess will show restricted diffusion.

**Fig. 14:**
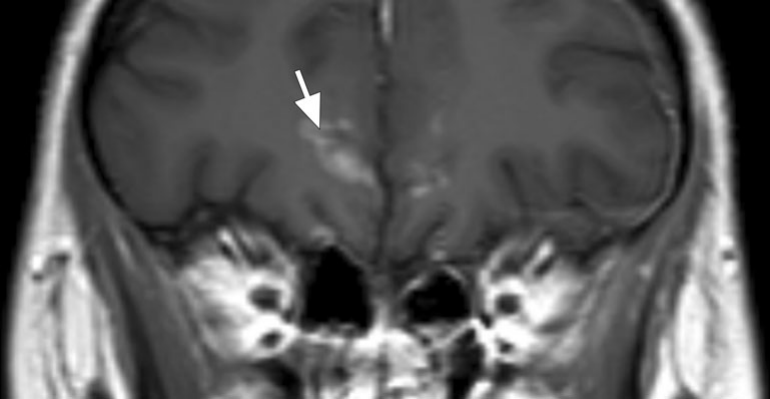
Leptomeningitis: coronal T1w ceMRI of a patient that developed subdural empyema due to frontal sinusitis (not shown). The image shows increased enhancement of the leptomeninges of the frontal lobes (arrow), indicating leptomeningitis

Meningitis is even more difficult to detect on CT, and MRI should be obtained if this diagnosis is sought for. At an early stage, MRI may be normal. On contrast enhanced images, abnormal meningeal enhancement may be seen of both pachymeninges and leptomeninges (Fig. 14). On FLAIR images a high signal extending into the sulci may be seen as a result of the thickened, oedematous meninges and arachnoid [21, 22, 35]. Meningitis may further be complicated by adjacent brain oedema, focal ischemic injury, and hydrocephalus [36]. Ischemia is best seen on DWI, showing restricted diffusion in the brain parenchyma. In some cases, purulent exudate can form in the subarachnoid space, which will also cause restricted diffusion [20].

## Conclusion

Complications arising from acute bacterial rhinosinusitis can result in life-threatening illness. Knowing the anatomic relationship of the paranasal sinuses to the orbital and intracranial compartment and the mechanisms of infectious spread, is paramount for early diagnosis of these complications. In addition, the radiologist needs to be aware of the specific imaging findings of orbital and intracranial complications of acute bacterial rhinosinusitis, including cavernous sinus thrombosis.
